# Lymphokine-activated killer cell susceptibility and adhesion molecule expression of multidrug resistant breast carcinoma

**DOI:** 10.1186/1475-2867-6-24

**Published:** 2006-11-03

**Authors:** Burhan Savas, Pauline E Kerr, Hugh F Pross

**Affiliations:** 1 Dept. of Oncology, Akdeniz University, Antalya, Turkiye; 2 Dept. of Microbiology and Immunology, Queen's University, Kingston, Canada

## Abstract

Reports showing susceptibility of multidrug resistant (MDR) cancer cells to immune effectors, together with P-glycoprotein (P-gp) expression in immune effector subsets, including immature natural killer (NK) cells, and some activated T cells, suggest P-gp or some changes associated with it, have implications in immune-mediated mechanisms. A series of experiments were done to determine the nature of alterations associated with susceptibility to immune effector cells of MDR tumor cells. A cell line isolated from the malignant pleural effusion of a breast cancer patient was transfected with human and murine MDR1 genes, and four variants with different levels of MDR were obtained. Lymphokine-activated killer (LAK) activity was measured by a ^51^Chromium release, and conjugate formation assays. MDR1 transfectant P-gp^+ ^breast carcinoma lines had increased LAK susceptibility compared to their parent line. Some part of the increased LAK susceptibility of drug-resistant cell lines was at the binding/recognition level as shown by conjugate formation assays. This suggests that differences may exist between paired cell lines with respect to the expression of cell adhesion molecules (CAMs). Monoclonal antibodies (mAbs) to CAMs and flow cytometry were used to quantitate these antigens. The CAMs studied were those previously found to be upregulated by stimulating NK cells with (interleukin-2) IL-2; ICAM-1 (CD54), LFA-3 (CD58), N-CAM (CD56), and the β chain of LFA-1 (CD18). Although no differences in these CAMs were found between the breast carcinoma line and its MDR1-transfected variants, the target susceptibility results given above suggest that IL-2 treatment could be effective in combination with current protocols using chemotherapeutics, monoclonal antibodies (mAbs) and stem cell transplantation.

## Background

Breast cancer is a serious health concern and a major public health challenge with an incidence of 217.500 and breast cancer deaths of 40.600 in USA being estimated in 2004 [1]. The primary treatment of early stage breast cancer is by surgery and radiation therapy, and a cure is frequently achieved if the cancer has not spread. The addition of chemotherapy can increase the long-term survival of patients with breast cancer, presumably by killing cancer cells beyond the operative field, and the use of chemotherapy after recurrence of breast cancer can provide significant palliation. However, post-chemotherapy recurrence of cancer that is resistant to further chemotherapeutic measures is a major problem. Low expression of the MDR1 and multidrug resistance-associated protein 1 (MRP1) genes is observed in primary breast cancers, however MDR1 expression is increased in tumors refractory to chemotherapy, in lobular breast carcinoma and in tumors from the patients below age 50, all of which are known to be associated with poor prognosis [2-4] The MDR1 gene frequently becomes amplified and its mRNA is overexpressed in cells selected for resistance to drugs such as doxorubicin, daunomycin, vinca alkaloids, taxanes, and actinomycin D. Anthracyclines such as doxorubicin, taxanes such as paclitaxel and the vinca alkaloids such as vinorelbine are very active agents used frequently in the treatment of breast cancer [5].

One aspect of breast cancer biology that hinders cure is tumor cell heterogeneity and phenotypic variability. Some tumor cells may be killed by chemotherapy, but the ability of a tumor cell population to adapt to a new environment results in progression of the disease. This arises from either the tumor cells that are already resistant to the therapeutic drug or tumor cells that develop resistance during the course of the treatment.

After the impressive progress in the understanding of the basic tumor biology of the breast cancer, demand for additional markers of biologic relevance for the prediction of breast cancer outcome and of response to various chemotherapy, radiotherapy and immunotherapy regimens has increased in the recent years.

Over the ensuing decade, systemic administration of c-erbB-2 monoclonal antibody trastuzumab in patients with c-erbB-2 over-expressing tumors was found to produce objective regressions in 11 to 26 percent of patients with metastatic breast cancer [6,7]. Recently, four large multicenter designed to test the role of trastuzumab as adjuvant therapy after surgical treatment of primary breast cancer found an absolute benefit of 6 to 8 percent at two years and 18 percent by four years [6,8,9]. This is particularly impressive given the evidence that c-erbB-2 gene and protein abnormalities in breast cancer predict poor prognosis and resistance to tamoxifen therapy and chemotherapy regimens such as CMF (cyclophosphamide, methotrexate, 5-fluorouracil) [7,10] However, the amplification and overexpression of c-erbB-2 proto-oncogene has been described in approximately 25–30 % of breast carcinomas [11,12].

LAK cell immunotherapy has been shown to have the potential to eradicate P-gp^+ ^MDR tumor cell populations in ovarian, SCLC, malignant melanoma and renal cell carcinoma [13-16]. In addition to P-gp^+ ^MDR tumor cell populations, c-erbB2(+) positive breast cancer cells may be eradicated to a better extent with c-erbB2 mAb + LAK cells since LAK cells have strong antibody-dependent cell-mediated cytotoxic (ADCC) activity [16].

The objectives of the current study were:

1. To quantitate the LAK cell-mediated lysis (CML) susceptibility of drug sensitive and drug resistant breast cancer cell lines.

2. To study the binding/recognition step of LAK CML in these cell lines and its contribution to differences in LAK CML of the paired sensitive and MDR cell lines.

3. To measure the level of expression of CAMs shown to be involved in the binding/recognition step of lysis and to compare these levels within the paired cell line groups.

## Results

### Human LAK cell-mediated lysis

Peripheral blood lymphocytes collected from normal volunteers were activated with human recombinant interleukin 2 and tested for LAK cell activity against the drug sensitive human breast carcinoma cell line MDA-MB-231, and its MDR-transfected variants MPAM-26, MPAHS-1-10, MPAHS-1-300, and MPAHS-DOX150. LAK cell cytotoxicity was also tested against the NK-sensitive, LAK-sensitive cell line K562, and the NK-resistant, LAK-sensitive RAJI cell line. LAK cell-mediated cytotoxicity against target cells was evaluated by an 18 hr ^51^Cr-release assay.

**Figure 1:**
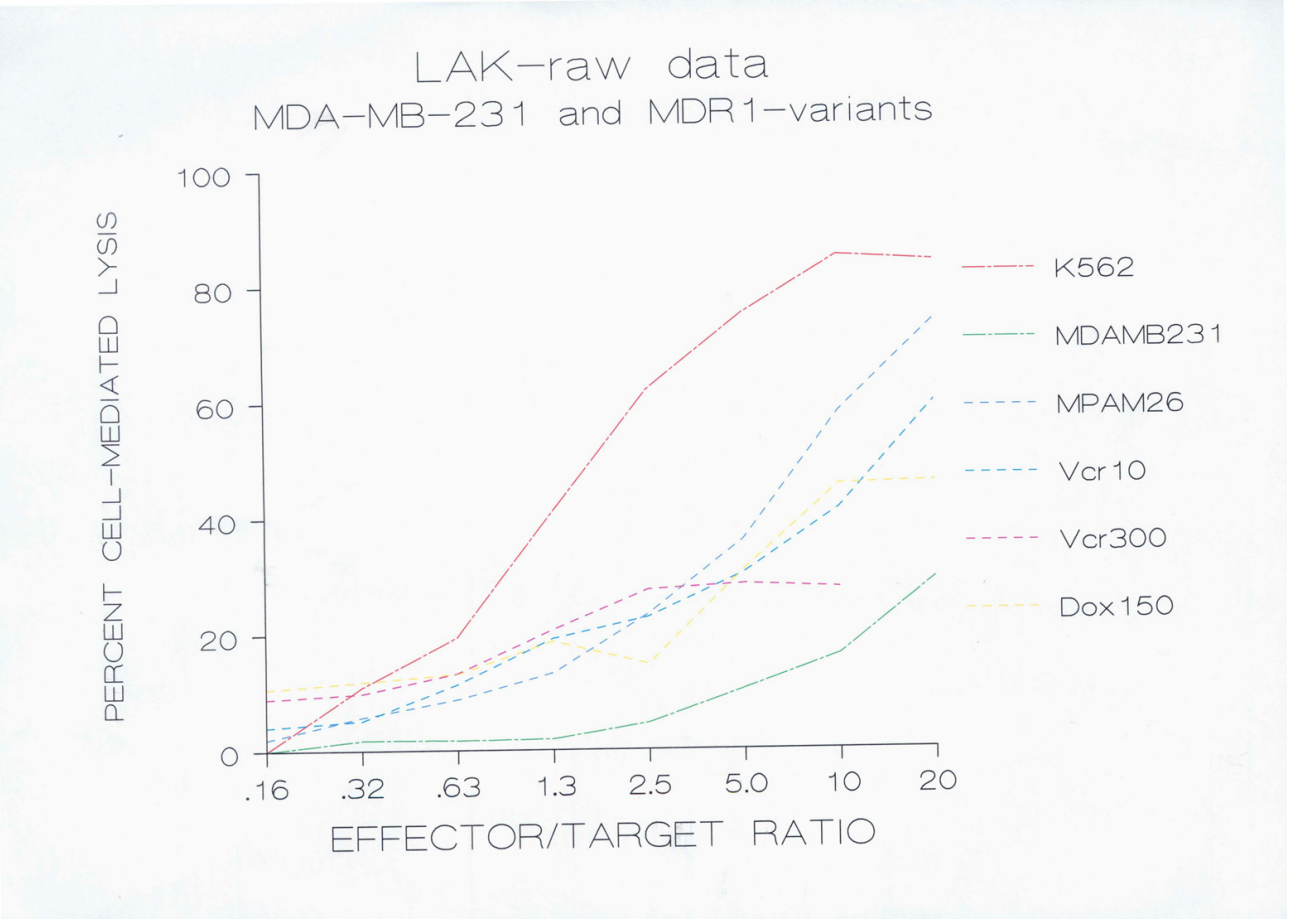
**Susceptibility of MDA-MB-231 breast carcinoma cell lines to human LAK CML (^51^Chromium-release data)**. 2 × 10^6 ^PBMC/ml were incubated with 1000 U/ml human rIL-2 in RPMI 1640 containing 10% autologous plasma in a total volume of 10 ml. After 5 days, these cells were harvested and set up in an 18 hr ^51^Cr-release assay to measure the cytotoxicity against MDA-MB-231 (drug-sensitive breast carcinoma cell line), MPAM-26 (murine MDR-1 transfected), and Vcr10 (MPAHS-1-10), Vcr300 (MPAHS-1-300), Dox150 (MPAHS-DOX150), human P-gp transfected multidrug resistant variants of MDA-MB-231. Percent specific cell-mediated lysis was plotted against effector/target ratio based on data obtained from one experiment representative of 15 independent experiments performed by three independent investigators. The data from the 15 experiments were converted to lytic units and tested for significant difference (see text).

**Figure 2:**
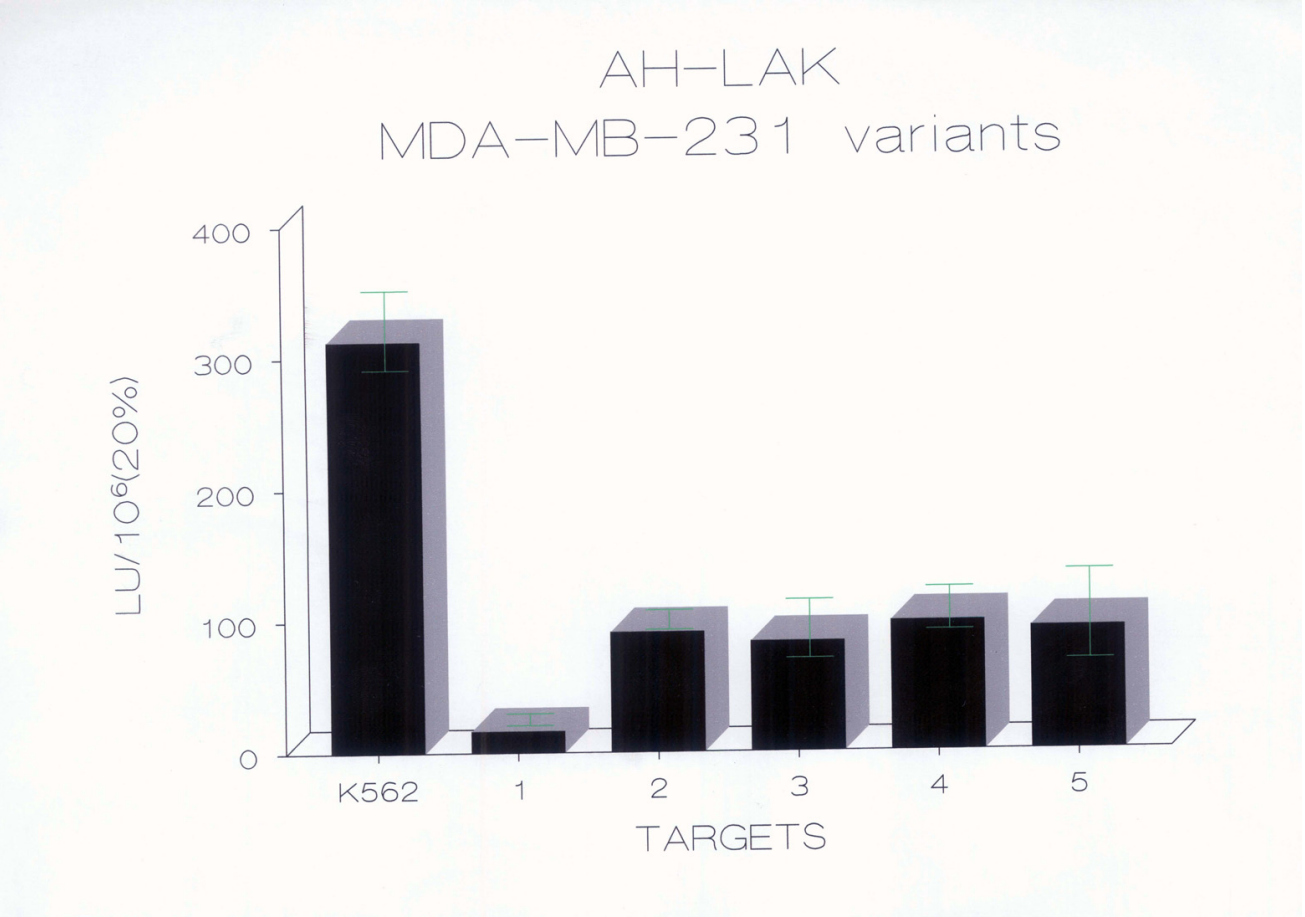
**Human LAK cell sensitivity of MDA-MB-231 breast carcinoma cell lines expressed as LU**. Using the same experimental design as in Fig. 1, results obtained from LAK CML experiments with MDA-MB-231 breast carcinoma cell lines are demonstrated as lytic units ± standard deviation. 1 = MDA-MB-231 (drug-sensitive breast carcinoma cell line), 2 = MPAM-26 (murine P-gp transfected variant of MDA-MB-231), 3 = MPAHS-1-10 (human P-gp transfected variant of MDA-MB-231), 4 = MPAHS-1-300 (human P-gp transfected variant of MDA-MB-231),5 = MPAHS-DOX150 (human P-gp transfected variant of MDA-MB-231). The results shown are from an experiment representative of 15 independent experiments, performed by three independent investigators. LAK effectors were from three different donors. Results are expressed as LU/10^6^. Each lytic unit value is derived from 8 effector/target (E/T) ratios as described in the Methods.

The percent chromium release, lytic unit values and cytotoxicity relative to the parental line were calculated in order to determine if there were differences in LAK cell susceptibility between paired cell lines. The susceptibility of MDA-MB-231 and its MDR variants to LAK cell lysis is shown in Fig. 1 as percent cell-mediated lysis (or percent specific ^51^Cr release), and in Fig. 2 as lytic units.

In these experiments, the murine MDR1-transfected variant MPAM-26 and the human P-gp^+ ^transfected lines MPAHS-1-10, MPAHS-1-300 and MPAHS-DOX150 were found to have a four to five fold increased sensitivity to LAK CML in comparison to the drug sensitive parent line MDA-MB-231. Lysis of MPAM-26 was significantly higher than that of MDA-MB-231, as shown by both Student's paired "t" test (p = 0.04, 1-sided) (log-transformed data) and the Wilcoxon signed-ranks matched-pairs non-parametric test (p = 0.02, 2-sided). The correlation between the two sets of paired data was r = 0.95 for the log-transformed data. Lysis of MPAHS-1-10 was significantly higher than that of MDA-MB-231, as determined by both Student's paired "t" test (p = 0.008, 2-sided) (log-transformed data) and the Wilcoxon signed-ranks matched-pairs non-parametric test (p = 0.003, 2-sided). The correlation between the two sets of paired data was r = 0.96 for the log-transformed data. Lysis of MPAHS-DOX-150 was also significantly higher than that of MDA-MB-231, as determined by the Wilcoxon signed-ranks matched-pairs non-parametric test (p = 0.03, 2-sided), and by the Student's paired "t" test (p = 0.05, 1-sided) (log-transformed data). All of the above statistics were calculated with n = 15.

### Effect of P-gp expression on ^51^Cr efflux

P-gp is postulated to function as an energy-dependent efflux pump in MDR resistant cells. Therefore, ^51^Cr might be actively pumped out of MDR cells during the ^51^Cr-release assay by the P-gp. This might complicate the interpretation of changes in the susceptibility of P-gp^+ ^cells to LAK CML. In order to examine whether ^51^Cr is actively pumped out of MDR cells by P-gp during the assay, the "spontaneous" release (SR) of ^51^Cr from MDR breast cell lines and their sensitive counterpart was determined (Table 1). The SR value represents the release of ^51^Cr from labelled cells incubated in medium alone (without LAK effector cells) for the duration of the assay. MDR carcinoma cell lines exhibited unchanged SR with respect to their sensitive counterparts. It is concluded that, in general, P-gp expression was not associated with any significant change in spontaneous ^51^Cr release during the time of experiment. These results are in agreement with similar results for the effect of P-gp expression on ^51^Cr efflux in MDR SCLC and ovarian carcinoma cell lines [15,16].

**Table 1:** Spontaneous Release (SR) exhibited by MDR and drug-sensitive breast carcinoma cell lines

**Cell Line**	**SR**	**SE**	**n**
MDA-MB-231	16.1	3.3	8
MPAM 26	17.1	6.6	10
MPAHS-1-10	17.0	1.9	8
MPAHS-1-300	19.2	4.2	8
MPAHS-DOX150	18.7	5.7	8

SR = Spontaneous ^51^Cr release during 18 hour assay in wells which contain targets, but not LAK cells. SR is expressed as % of the maximum.

SE = Standard error. n = Number of independent experiments

### Effect of P-gp expression on LAK cell-mediated cytotoxicity at the binding/recognition level

**Figure 3:**
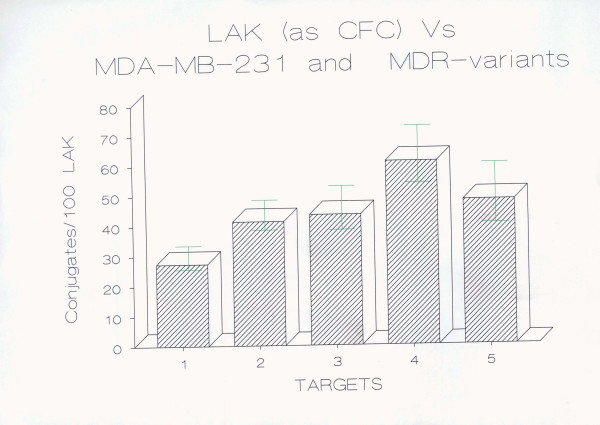
**LAK cell binding of MDA-MB-231 breast carcinoma cell lines as shown by conjugate formation assay**. 1 × 10^5 ^LAK cells (0.1 ml) and 1 × 10^5 ^target cells (0.1 ml) were mixed in the same tube, centrifuged for 2 min at 500 g and incubated at 37°C for 10 min. The cells stained with trypan blue were observed under phase contrast microscope. 2 LAK cells forming a conjugate with a live tumor cell is demonstrated in Fig. 4. A LAK cell was observed to form a conjugate with a blue-dead tumor cell is visible in Fig. 5. Two blue-dead tumor cells are also seen in this picture. The percentage of LAK cells attached to the targets and vice versa was counted in a modified Cunningham chamber. And the data represent the arithmetic mean ± standard deviation of the mean percent LAK cells conjugated to targets (LAK:Target) of 4 independent experiments. The percentage of LAK:Target cell conjugates formed by the MDR breast carcinoma cell lines was found to be higher than that formed by the drug-sensitive parent cell line MDA-MB-231. This increase was statistically significant at p < 0.05 for MPAM 26, MPAHS-1-10, and MPAHS-DOX150, and at p = 0.01 for MPAHS-1-300 (Student's paired "t" test, 1-sided). 1 = MDA-MB-231 2 = MPAM 26 3 = MPAHS-1-10 4 = MPAHS-1-300 5 = MPAHS-DOX-150

In order to examine whether the increased or decreased LAK susceptibility of MDR breast cell lines is at the binding/recognition or post-binding level, conjugate formation assays were performed. PBMCs were activated with human rIL-2 and tested for LAK cell binding activity against the breast carcinoma cell line MDA-MB-231 and its MDR variants MPAM 26, MPAHS-1-10, MPAHS-1-300, and MPAHS-DOX150. The data obtained from 4 separate experiments are shown in Fig. 3.

All of the MDR variants had increased LAK:Target binding that was statistically significant. The parent line had approximately 27% of LAK cells bound to it versus 41% to 61% for the MDR cell lines. MPAM 26, MPAHS-1-10 and MPAHS-DOX150 had 50% to 80% increased binding compared to the drug sensitive parent line and MPAHS-1-300 had approximately 120% increased binding. The results are in agreement with previous studies for the SCLC and ovarian carcinoma cell lines [15,16]. Increased LAK:target conjugation of MDR cell lines compared to drug-sensitive parents suggest that the increased susceptibility to LAK CML of these MDR cell lines, compared to its drug sensitive parental counterpart, is due mainly to greater binding/recognition.

### Adhesion molecule expression and correlation with LAK CML

In order to determine whether or not differences at the binding/recognition step of LAK CML are associated with differences observed in the LAK susceptibility of drug sensitive and resistant paired breast cell lines, the level of surface expression of adhesion molecules ICAM-1, LFA-3, N-CAM, and CD18, as well as P-gp and HLA Class I were measured in conjunction with the LAK CML of the cell lines. The level of expression of the surface molecules was measured using commercially available mAbs and flow cytometric analysis. Data are expressed in terms of percent positive cells and monoclonal antibody binding sites values calculated as described in the Methods.

There was no significant difference in the expression of CAMs or HLA Class I antigen between the drug sensitive parent line MDA-MB-231 and its MDR1-transfected counterparts. Differences were observed in the level of P-gp expression. P-gp determined by MRK16 mAb was not expressed on the parent line or on murine MDR1-transfected MPAM 26. However, when MDA-MB-231 and MPAM-26 cells were stained with mAb JSB-1 which, unlike MRK16, recognizes a conserved cytoplasmic epitope of P-gp [17], P-gp expression on MPAM-26 was observed (data not shown).

MPAHS-1-300 and MPAHS-DOX150 had significantly increased levels of P-gp compared to the parent line (p < 0.001, Student's "t"-test, 2-sided), and MPAHS-1-10 was higher at p = 0.03 (Student's "t"-test, 1-sided). Relative Fluorescence Intensity (RFI) values were calculated according to the equation : RFI = RFI (test sample) – RFI (negative control).

Samples were prepared by incubating the cell lines with each mAb listed followed by FITC-labeled secondary Ab. Samples were read on a Becton Dickinson FACS IV. Anti-CD54 (ICAM-1), anti-CD58 (LFA-3), anti-CD56 (N-CAM) or Leu19, anti-CD18, which is the β chain of LFA-1, MAC-1 and P150/95 were the anti-CAM mAbs studied. Levels of anti-P-gp mAb MRK16, which labels the surface epitope of P-gp, and W6.32 (anti-HLA-A,B,C) were also measured.

The percentage of cells expressing surface antigen was determined using flow cytometric analysis. A lower gate channel was set on the negative control sample so that 5% of the total cells lay above the gate. The same gate was set on each test sample, and the percentage of cells which lay above the gate was determined. This number, after 5% was subtracted from it, was reported as the percent positive cells in each test sample. The data are shown in Table 2. The percent positive cells was directly related to the RFI of the positive cell population.

**Table 2:** P-gp and adhesion molecule expression of MDR1-transfected human breast carcinoma cell lines as percent positive cells, and mAb binding sites (× 10^4^)

	MDA-MB	MPAM-26	MPAH1-10	MPAH1-300	MPAHDOX
MRK16 = A	12.07	8.52	30.57	56.47	78.50
s.d.	4.10	1.62	10.85	3.20	9.68
					
MRK16 = B	5.45	3.00	4.41	12.85	38.36
s.d	0.21	0.56	0.35	2.79	10.23

ICAM-1 = A	58.65	59.39	62.63	55.13	76.36
s.d.	1.79	2.03	4.56	7.44	1.41
					
ICAM1 = B	16.43	13.36	12.66	19.00	35.54
s.d.	1.20	3.48	4.80	8.81	12.25

LFA-3 = A	82.37	83.12	83.30	84.12	89.38
s.d.	2.31	1.27	6.71	4.84	1.42
					
LFA-3 = B	23.65	24.65	18.08	33.22	53.66
s.d.	11.36	3.31	3.41	6.20	9.64

CD18 = A	2.44	0.13	9.69	18.43	0.17
s.d.	1.35	3.52	2.52	3.26	0.93
					
CD18 = B	7.44	5.66	5.24	7.77	7.58
s.d.	1.31	1.72	1.35	1.57	1.76

CD56 = A	3.72	0.58	4.18	0.44	0.33
s.d.	2.13	0.44	5.88	0.75	0.57
					
CD56 = B	6.92	4.90	3.97	5.97	5.93
s.d.	0.91	1.20	0.81	1.29	0.07

A = Percent positive cells

B = mAb binding sites (× 10^4^/cell)

### Quantitation of the Ab binding sites on cell surfaces

In order to assess the absolute quantitative level of expression of the different surface antigens studied, the number of actual Ab binding sites on the cell surface was determined. Differences exist between the number of secondary antibodies that may bind to a primary Ab and thus, differences exist in the measure of fluorescence obtained. In order to make accurate comparisons between the expression of different surface antigens and to obtain values that are consistent and comparable between laboratories, it is necessary to correct for this difference. In order to accomplish this, a microbead quantitation system was used. After the standardization of the FACS with calibrated fluorescence microbeads, the effective fluorescence-to-protein ratios for each mAb are calculated. Values obtained from three separate experiments for the MDA-MB-231 breast carcinoma cell line series are presented in Table 2 (above – "B" rows).

## Discussion

In this study, the following observations were made:

1. Three human MDR1-transfected, and one mouse MDR1-transfected breast carcinoma cell lines had increased sensitivity to LAK cell-mediated lysis compared with the parental line. The increases of these targets were statistically significant.

2. There were no significant differences in the expression of adhesion molecules ICAM-1, LFA-3, CD18, and CD56 or HLA Class I antigen between drug-sensitive and MDR breast carcinoma paired cell lines.

3. The MDR breast tumor cell lines demonstrated increased binding to LAK cells in comparison to the parent line.

Immunotherapy using LAK cells is a candidate treatment modality which may be effective in patients with tumors that are resistant to chemotherapy in breast, ovarian, and colon carcinoma, small cell lung cancer and melanoma [14-16,18-20]. There is a need for more studies on LAK immunotherapy assessing the susceptibility of tumors to LAK CML and determining predictive factors for the potential success of immunotherapy. Previous studies by us and others in the literature (for a review please see Table 2 of reference paper [14]) suggest that P-gp expression or various changes associated with P-gp increase the susceptibility of MDR tumor cell lines to LAK cell mediated cytotoxicity. After observing the increased lymphokine-activated killer (LAK) cell sensitivity of the multidrug resistant (MDR) P-gp(+) small cell lung carcinoma line H69/LX4, but not the multidrug resistant-associated protein(+), P-gp(-) and glutathione-S-transferase pi (+) variant H69/AR compared to the parental line H69 [15], we chose to investigate LAK cell susceptibility of mouse and human multi-drug resistance 1 (MDR1) gene transfected MDR cell lines. Since the MDR cell line variants of SCLC model in the article above were developed by stepwise increments of chemotherapeutics, there is a concern that the results might have been affected from various changes other than P-gp that the cell line variants might have acquired during this lengthy period. In order to assess this possibility, we analyzed 3 human MDR1-transfected and one mouse MDR1-transfected variants of a drug-sensitive breast cancer line MDA-MB-231 in this paper.

In order to eliminate factors other than P-gp that may develop during the long time necessary for a cell line to develop P-gp^+ ^MDR during incremental drug challenge of parental drug sensitive cell lines; human and murine MDR1 cDNA were transfected into a metastatic breast carcinoma cell line and P-gp was allowed to demonstrate itself under the selecting pressure of 3 different drugs at four different concentrations in a short time compared to the previous MDR lines developed without MDR1 transfection. Increased LAK susceptibility was observed in all of the four MDR1-transfected cell lines. It should be emphasized that increased LAK susceptibility was observed for both human and murine P-gp expressing targets. Previously, increased human LAK effector killing against P-gp^+ ^Chinese hamster ovary target cell lines was also shown [16]. In the same study increased LAK susceptibility of human P-gp expressing tumor targets against murine LAK cells was demonstrated in a similar fashion. These results suggest increased LAK susceptibility for P-gp (+) targets-crossed species barriers for both effectors and targets among mammals.

Could this increased LAK susceptibility of P-gp^+ ^cells be due to a change in adhesion molecules? CAMs may bind either to self (homophilic adhesion) or to other ligands (heterophilic adhesion). After in vitro incubation with IL-2, human NK cells demonstrated four- to sixfold increases in surface levels of CD18, CD54, CD58, and CD56 [21]. Furthermore, essentially all NK cells became CD54^+ ^within 3 days of exposure to IL-2. In addition, IL-2 dramatically increased the levels of CD56 and CD58 on NK cells. In contrast, T cells did not demonstrate comparable up-regulation of these CAMs after incubation with IL-2. Increases in NK cell CAM expression were associated with enhanced formation of effector to target cell (E/T) conjugates, enhanced killing of NK-sensitive targets, and the induction of cytotoxicity for previously NK-resistant targets (LAK activity).

In 1991, a new association between P-gp expression in invasive colon carcinoma cells and increased metastases to lymph nodes was described [22]. Although the adhesion profiles of P-gp have not been examined in detail, several observations raised the possibility that P-gp may influence cell adhesion and, as a result of this, the process may be involved in cancer dissemination and possibly, the sensitivity of tumor cells to LAK CML. Similarly, P-gp expression was accompanied by increased homotypic adhesion in epidermoid carcinoma cells in tissue culture [23,24]. In 1990, Grogan et al. observed a relationship between the hyperexpression of both P-gp and N-CAM molecules in myeloma plasma cells in tissue culture [25]. These cells showed increased homotypic adhesion. However, N-CAM and P-gp co-modulation was not observed in our study (Table 2).

MHC, ICAM-1, LFA-3, NCAM and CD18 were examined in MDR1-transfected breast carcinoma cell lines in this study, and no significant correlations were found for the breast carcinoma cell lines neither between LAK CML and these CAM expressions, nor between LAK CML and MHC Class I molecule expression. Similar results were also observed previously in MDR P-gp^+ ^the ovarian carcinoma cell line 2780.AD645 and SCLC line H69/LX4 [15,16].

Another possibility for the mechanism may involve gangliosides. In 1991, Mazzoni et al. found an involvement of cell membrane gangliosides in the development of the A2780-Doxorubicin MDR phenotype, and suggested a possible involvement of the ganglioside GD1a in the increased sensitivity of MDR cells to immuno-mediated cytolysis [26]. Indirect mechanisms related to the biochemical alterations occurring in MDR neoplastic cells may influence LAK CML susceptibility. A significant increase of Ca++/phosphokinase C (PKC) activity, frequently present in MDR cells [27] could enhance the cytotoxic effects of cytokine(s) released by activated lymphocytes, since such a release is preceded by an influx of Ca^++ ^and PKC involvement [28]. Alterations of membrane fluidity due to phospholipid changes associated with P-gp expression in MDR tumor cells may lead to fragility of the cells, resulting in increased susceptibility to released cytotoxins [29,30].

The differentiation stage of tumor cells could also influence susceptibility of target cells. A decrease of NK sensitivity is detectable after induction of a more differentiated phenotype in colon-carcinoma lines by treatment with differentiating agents [31] or by transfection with oncogenes [32]. The expression of adhesion or adhesion-like molecules and the differentiation stage of the target cells appear to be related to each other [20]

For MDR1-transfected breast carcinoma cell lines, the increased binding of MDR cell lines in comparison to the sensitive parent line for LAK:Target binding suggests that differences in the LAK cell-mediated lytic susceptibility of these cells are due in part to differences at the binding/recognition level.

The possible increased LAK susceptibility and binding of P-gp(+) MDR metastatic breast cancer cells as suggested in this paper encourages studies toward mAbs and LAK cells for the treatment of breast cancer patients since LAK cells have strong ADCC. The development of "designer" mAbs, chimeric mAb to particular antigens and receptors, and their potential to target cancer cells and enhance their susceptibility to the effector arm of the immune system are currently providing important new opportunities. The potential for the use of monoclonal antibodies such as P-gp and C-erb-B2 for the better therapeutic efficacy of LAK immunotherapy in breast cancer is particularly encouraging.

## Conclusion

Our results demonstrated that four MDR1-gene transfected metastatic breast carcinoma cell lines have increased susceptibility and binding to the lymphokine-activated killer cells. Interleukin-2 administration seems to be a promising chemoimmunotherapy improving modality in MDR1 over-expressing tumors.

## Methods

### Media and reagents

Unless otherwise indicated, media used to culture cell lines consisted of RPMI 1640 containing 10% FCS supplemented with 10 mM L-glutamine (Gibco/BRL, Grand Island, NY). Media used for the generation of LAK cells consisted of culture media without FCS, supplemented with 10% autologous plasma. Recombinant IL-2 was used at a concentration of 1,000 U/ml (Cetus Corp., Emeryville, CA), which had been pre-determined to yield maximum LAK cell generation. Vincristine and doxorubicin were purchased from Sigma, St. Louis, MO. Geneticin was purchased from Gibco/BRL.

### Monoclonal antibodies

Anti-CD54 (ICAM-1) and anti-CD58 (LFA-3) were purchased from AMAC Inc., Westbrook, ME, anti-CD56 (N-CAM) was purchased from Becton Dickinson, Mountain View, CA, and anti-CD18 (β chain of LFA-1) was purchased from Dakopatts, Glostrup, Denmark. Anti-P-gp mAb MRK16, which labels the first extracellular domain of P-gp which is heavily N-glycosylated and shows the least homology between human and murine counterparts [33] was a generous gift from Dr. T. Tsuruo, Hoechst, Japan. Therefore MRK16 mAb is only reactive with P-gp of human class I isoform (MDR1), but not with the human class III isoform, or other classes of P-glycoproteins from hamster, mouse, monkey, horse, pig, cow, and rabbit [34]. The negative control used was Coulter anti-IgM (Coulter, Hialeah, FL). The secondary antibody used was the F(ab')_2 _fragment of goat anti-mouse Ig (IgG, IgA, IgM) (Cappel Labs, Cooper Biomedical Inc., Westchester, PA).

### Preparation of peripheral blood lymphocytes (PBL) and LAK cell generation

Venous blood was collected from healthy donors in heparinized Vacutainer tubes. Mononuclear cells were prepared by centrifugation (850 g, 15 min) over Ficoll-Isopaque and were depleted of monocytes by incubation on autologous plasma coated plastic dishes for one hour at 37°C. These cells were recognized as PBL and were used in NK studies. PBL (1–2 × 10^6^/mL) were incubated in 25 cm^3 ^cell culture flasks (Corning) for 5–8 days in a 5% CO_2 _environment at 37°C with RPMI medium containing 10 mM L-glutamine, 10% autologous serum and 1000 U/mL human recombinant IL-2, and were used as LAK effectors [35]. LAK cells harvested after this procedure is demonstrated in LAK cells file ' [see Additional file 1]'.

### Cell lines and transfection

The human poorly-differentiated, estrogen receptor-negative and hormone-insensitive metastatic breast carcinoma cell line MDA-MB-231 (ATCC HTB26), which was isolated from pleural metastases of a woman with breast cancer [36,37] was transfected with murine or human P-gp cDNA resulting in the drug resistant variants MPAM-26, MPAHS-1-10, MPAHS-1-300, and MPAHS-DOX150. Briefly, exponentially growing MDÁ-MB-231 cells were transfected with PSKGA, which has a full length human MDR1 cDNÁ, and MDR1 cDNÁ containing plasmiä pGEM-4(ATCC clone designation pHDR5A). according to calcium phosphate precipitation method [38]. Then, 16 hours later transfected cells were subcultured in selective medium containing 400 microgram/ml geneticin (G418, Gibco). After first selection, copy numbers of MDR1 gene were amplified under stepwise increasing concentrations of doxorubicin, and vincristine.

Cells were split using EDTA (Gibco/BRL) at 80% confluence. Cells were fed 2 times a week. The continuous cell line drug supplements were as follows: MPAM-26 G418 (Geneticin, Gibco) 400 μg/ml; MPAHS-1-10 Vincristine Sulfate (Sigma) 10 ng/ml; MPAHS-1-300 Vincristine Sulfate (Sigma) 300 ng/ml; MPAHS-DOX150 Doxorubicin Hydrochloride (Sigma) 150 ng/ml.

K562 (NK-sensitive, LAK-sensitive standard) is a human erythroleukemia cell line isolated from a pleural effusion of a patient with chronic myelogenous leukemia in blast crisis [39]. K562 was obtained from the ATCC (# CCL 243, Rockville, MD). All cell lines were maintained by continuous culture as described, and periodically tested for mycoplasma contamination by the DAPI (4',6-Diamidino-2-phenylindole) (Sigma Chemical Co.) fluorescence assay [40].

### Cell line treatment prior to experimentation

In order to create similar conditions for the comparison of the cytotoxic cell susceptibility and adhesion molecule expression of drug-sensitive *vs *drug-resistant pairs, breast target cell lines were resuspended at a concentration of 0.3–0.6 × 10^6 ^cells/flask in new flasks 5 days prior to the experiments in drug-free medium. The culture medium was changed each of the 5 days, and the experiments were done while the target cells were in the exponential growth phase.

### Cytotoxicity assays

LAK activity was assessed in an 18 hr ^51^Cr release assay [41] against the standard target K562, and MDA-MB-231 (human adenocarcinoma breast, P-gp^-^), MPAM 26 (murine MDR1-transfected), MPAHS-1-10, MPAHS-1-300, MPAHS-DOX150 (human MDR1-transfected). Single cell suspensions of target cells in a logarithmic growth phase were labelled with ^51^Cr. Effector/target cell concentrations were adjusted to 40:1, 20:1, 10:1, 5:1, 2.5:1, 1.25:1, 0.62:1, and 0.31:1 in 96-well microtiter plates, then incubated for 18 hours at 37°C. The plates were then centrifuged (200 g, 10 min), and supernatants from each well were counted in a Beckman 5500 gamma-counter.

Percent specific release of each target was calculated with following formula.

% specific release=cpm (test) - cpm (spontaneous release)cpm (maximum) - cpm (spontaneous release)×100
 MathType@MTEF@5@5@+=feaafiart1ev1aaatCvAUfKttLearuWrP9MDH5MBPbIqV92AaeXatLxBI9gBaebbnrfifHhDYfgasaacH8akY=wiFfYdH8Gipec8Eeeu0xXdbba9frFj0=OqFfea0dXdd9vqai=hGuQ8kuc9pgc9s8qqaq=dirpe0xb9q8qiLsFr0=vr0=vr0dc8meaabaqaciaacaGaaeqabaqabeGadaaakeaacqGGLaqjcqqGGaaicqqGZbWCcqqGWbaCcqqGLbqzcqqGJbWycqqGPbqAcqqGMbGzcqqGPbqAcqqGJbWycqqGGaaicqqGYbGCcqqGLbqzcqqGSbaBcqqGLbqzcqqGHbqycqqGZbWCcqqGLbqzcqGH9aqpdaWcaaqaaiabbogaJjabbchaWjabb2gaTjabbccaGiabcIcaOiabbsha0jabbwgaLjabbohaZjabbsha0jabcMcaPiabbccaGiabb2caTiabbccaGiabbogaJjabbchaWjabb2gaTjabbccaGiabcIcaOiabbohaZjabbchaWjabb+gaVjabb6gaUjabbsha0jabbggaHjabb6gaUjabbwgaLjabb+gaVjabbwha1jabbohaZjabbccaGiabbkhaYjabbwgaLjabbYgaSjabbwgaLjabbggaHjabbohaZjabbwgaLjabcMcaPaqaaiabbogaJjabbchaWjabb2gaTjabbccaGiabcIcaOiabb2gaTjabbggaHjabbIha4jabbMgaPjabb2gaTjabbwha1jabb2gaTjabcMcaPiabbccaGiabb2caTiabbccaGiabbogaJjabbchaWjabb2gaTjabbccaGiabcIcaOiabbohaZjabbchaWjabb+gaVjabb6gaUjabbsha0jabbggaHjabb6gaUjabbwgaLjabb+gaVjabbwha1jabbohaZjabbccaGiabbkhaYjabbwgaLjabbYgaSjabbwgaLjabbggaHjabbohaZjabbwgaLjabcMcaPaaacqGHxdaTcqaIXaqmcqaIWaamcqaIWaamaaa@AAA5@

cpm = radioactivity count per minute in each well.

#### Data processing

^51^Cr release data were expressed in lytic units (LU) calculated according to the equation: y = A(1-e^-kx^) where y = percent of chromium release, x = Effector/target cell ratio, A = Maximal lysis by effector cells, k = a constant proportional to effector cell number, and equal to the negative slope of the target survival curve obtained by plotting ln (A-y) vs x [41]. Statistical significance analysis was done by using the SPSS program.

### Fluorescence activated cell sorter analysis

0.25–0.5 × 10^6 ^cells were placed in 12 × 75 mm test tubes (Falcon Labware Inc., Oxnard, CA) and centrifuged at 300 g for 6 min. The supernatant was removed and the mAb was added, tubes were vortexed and then incubated on ice in the cold room for 30–45 min. The concentrations of mAb added were: anti-ICAM-1 at 20 μl/test (5 × 10^5 ^cells/test), anti-LFA-3 at 10 μl/test (5 × 10^5 ^cells/test), anti-N-CAM at 20 μl/10^6 ^cells, anti-CD18 at 20 μl of 206 μg/ml stock, MRK16 at 10 μg/ml (5 × 10^5 ^cells) and W6.32 (ascites) diluted 1:500 (5 × 10^5 ^cells). Following this they were washed twice by centrifugation at 300 g for 6 min. with ice cold wash medium containing RPMI, 5% FCS and 1% sodium azide (Sigma). 100 μl of GAM-FITC (goat-antimouse Ig conjugated to FITC) (12.5 μg/ml) was added to the cell button, vortexed and incubated on ice for 30–45 min. Tubes were then washed 3 times at 300 g for 6 min. with cold wash medium (containing 1% azide). Finally, cells were resuspended in 0.5 ml RPMI (no FCS) and 1% paraformaldehyde (Sigma). Samples were analyzed on a Becton Dickinson FACS IV instrument, using an argon-ion laser running at 350 mW with the wavelength of excitation set at 488 nm and a 530/30 bandpass emission filter. Histograms were displayed on a 256-channel log scale. Uniform cell-sized quantitative fluorescent microbead standards (Simply Cellular R, Flow Cytometry Standards Corp., Research Triangle Park, NC) were used to calibrate the instrument. The percentage of cells expressing surface antigen was determined using flow cytometric analysis. In the analysis of the flow cytometry data, a lower gate channel was set on the negative control sample so that 5% of the total cells lay above the gate. The same gate was set on each test sample, and the percentage of positive cells which lay above the gate was determined. This number, after 5 % of the negative population was subtracted from it, was reported as the percent positive cells in each test sample. The mean log fluorescence channel was determined for the cells which lay above the gate channel. Further details are described in the Results section. The percent positive cells was related to the relative fluorescence intensity of the positive cell population.

### Quantitation of the number of mAb binding sites on target cells

The level of target cell surface antigens was quantitated by determining the number of antibodies bound by measuring the effective fluorescence/protein (F/P) ratios. The F/P ratio (as determined by absorbance) is the number of fluorescein molecules conjugated to an antibody molecule. The effective F/P ratio is the average fluorescence intensity in Molecules of Equivalent Soluble Fluorochrome (MESF) per antibody molecule. The FACS was calibrated for each experiment in terms of MESF per channel with the FCSC Quantum Kit (Flow Cytometry Standards Corp.). These standards consist of fluorescein molecules covalently bound to alkyl spacer groups on the exterior of uniform 5.8 μm hydrophobic polymeric microbeads. Their size and range of fluorescence intensity was optimized for analysis of common surface markers on peripheral blood lymphocytes. For each mAb studied, the fluorescence intensity in MESF was determined using the calibration curve and the peak fluorescence channel number obtained above. This MESF value was then divided by the number of binding sites on the microbead to give the effective F/P ratio.

Target cells were labelled and analysed on the FACS IV using the same indirect immunofluorescence technique as used for the microbeads. The mean channel number was used to obtain the MESF value again from the calibration curve, and this value was divided by the F/P ratio for the particular mAb to give the actual number of mAb binding sites on the target cell surface.

### Microscopic enumeration of LAK cell-target conjugates

Lymphocyte-target cell conjugates were analyzed visually using a modification of the method described by Pross [42]. IL-2 activated lymphocytes (1 × 10^6 ^in 1 ml) and target cells (1 × 10^6 ^in 1 ml) were mixed in ice-cold PBS and centrifuged at 200 g for 8 min in a 5 ml tube. After incubation for 25 min. on ice, the 1 ml cell pellet were removed, and gently resuspended. 50 μl conjugate suspension were mixed with 200 μl trypan blue, and an aliquot was transferred to a modified Cunningham chamber [42]. The percentage of conjugates was determined by counting the percentage of LAK cells attached to target cells using a phase contrast microscope. Dead cells stained with the trypan blue dye were also counted. Free lymphocytes, lymphocyte-target conjugates, free targets, and target-lymphocyte conjugates were counted for at least 200 lymphocytes.

## Competing interests

The author(s) declare that they have no competing interests.

## Authors' contributions

BS carried out the cell culture studies, and LAK cell generation-cytotoxicity-binding assays. PEK did the adhesion molecule profiles. HFP participated in the design of the study, statistical analysis, coordination and helped to draft the manuscript. All authors helped and approved the final manuscript.

**Figure 4:**
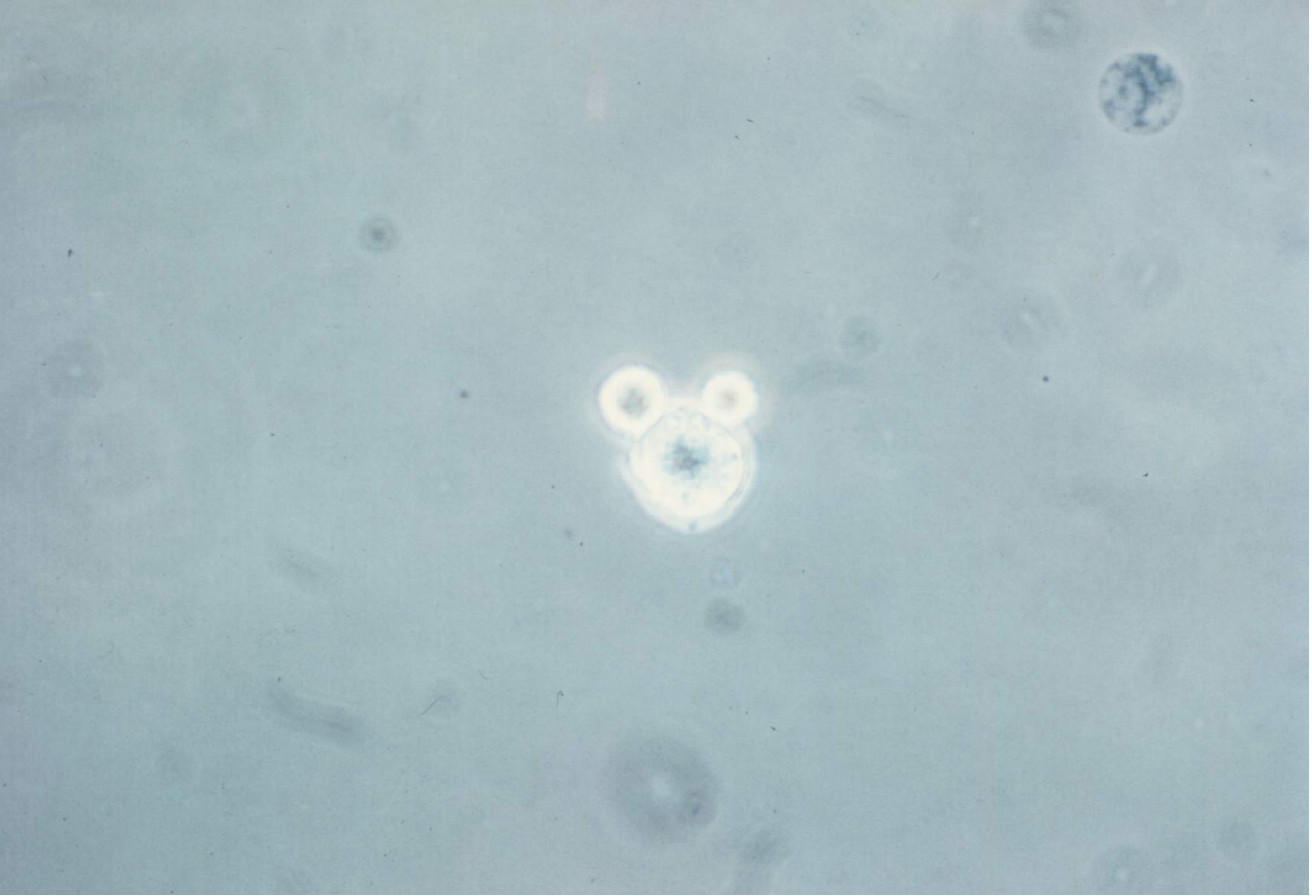
**LAK cell binding of MDA-MB-231 breast carcinoma cells**. 1 × 10^5 ^LAK cells (0.1 ml) and 1 × 10^5 ^target cells (0.1 ml) were mixed in the same tube, centrifuged for 2 min at 500 g and incubated at 37°C for 10 min. The cells stained with trypan blue were observed under phase contrast microscope. 2 LAK cells forming a conjugate with a live tumor cell is demonstrated.

**Figure 5:**
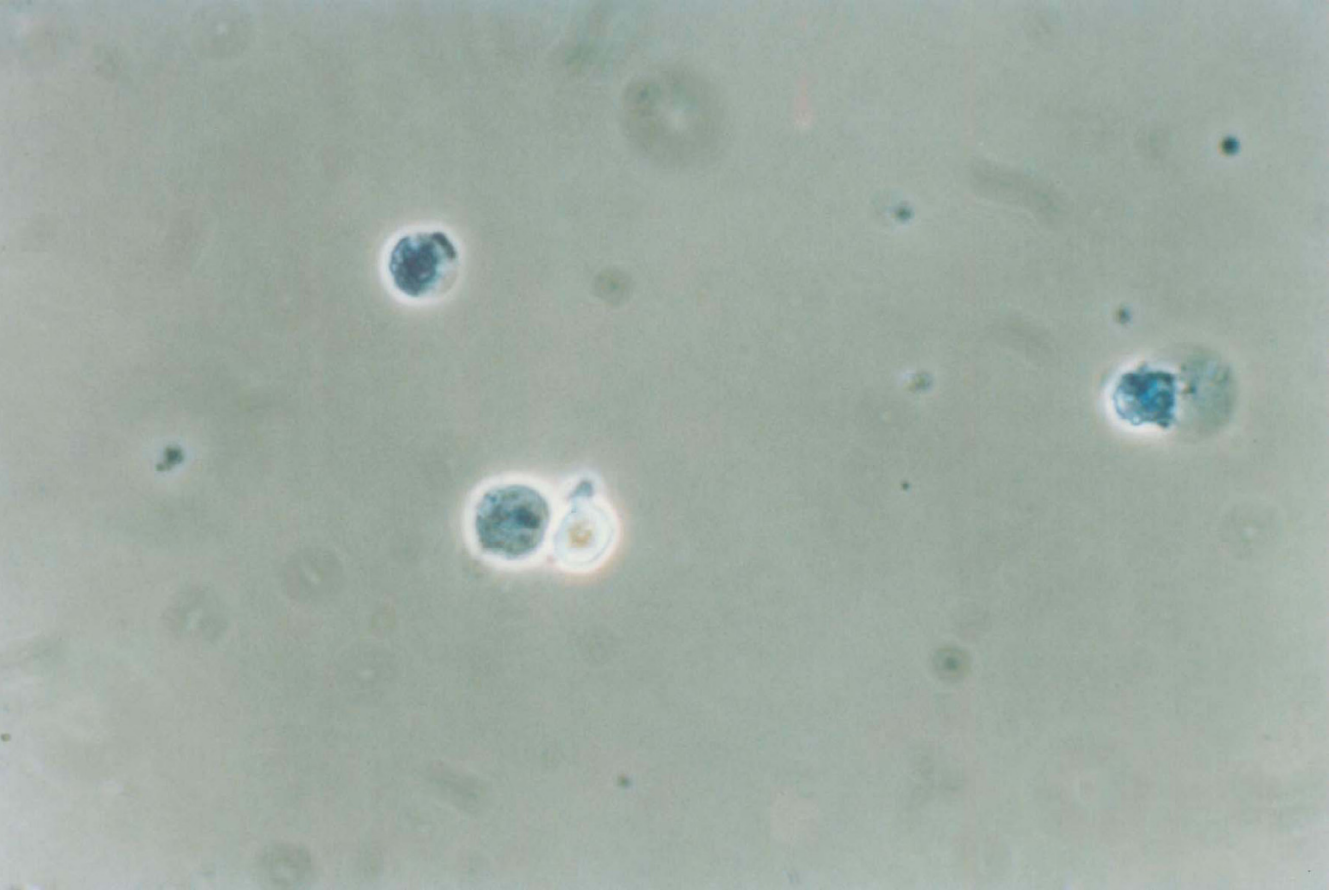
**LAK cell binding of MDA-MB-231 breast carcinoma cells**. 1 × 10^5 ^LAK cells (0.1 ml) and 1 × 10^5 ^target cells (0.1 ml) were mixed in the same tube, centrifuged for 2 min at 500 g and incubated at 37°C for 10 min. The cells stained with trypan blue were observed under phase contrast microscope. A LAK cell was observed to form a conjugate with a blue-dead tumor cell. Two blue-dead tumor cells are also seen in this picture.

## Supplementary Material

Additional file 1 LAK cells. Demonstration of lymphokine-activated killer cells colonies which were formed after 5–8 days incubation of PBL with human recombinant IL-2. Click here for file
