# Supplementary figures and images for: Lymphokine-activated killer cell susceptibility and adhesion molecule expression of multidrug resistant breast carcinoma
Source: Cancer Cell Int. 2006 Nov 3;6:24. doi: 10.1186/1475-2867-6-24 (PMC1635735; doi:10.1186/1475-2867-6-24)

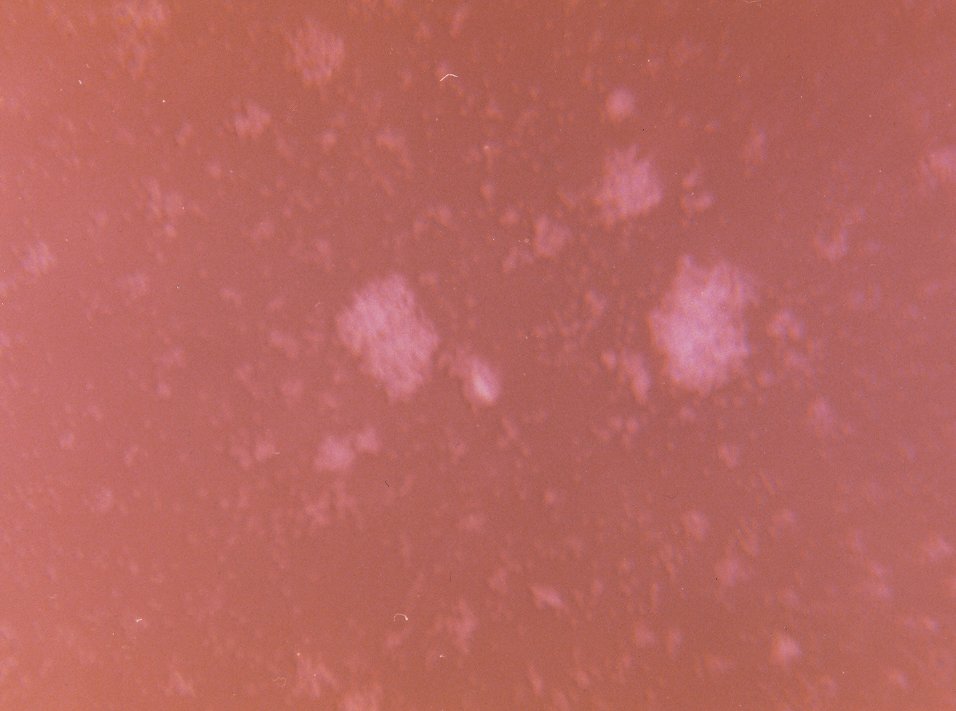

Supplement: Additional file 1 — LAK cells. Demonstration of lymphokine-activated killer cells colonies which were formed after 5–8 days incubation of PBL with human recombinant IL-2. [file 1475-2867-6-24-S1.doc]
